# Properties of Undoped Few-Layer Graphene-Based Transparent Heaters

**DOI:** 10.3390/ma13010104

**Published:** 2019-12-24

**Authors:** Yong Zhang, Hao Liu, Longwang Tan, Yan Zhang, Kjell Jeppson, Bin Wei, Johan Liu

**Affiliations:** 1 SMIT Center, School of Mechatronic Engineering and Automation, Shanghai University, Changzhong Road, Shanghai 201800, China; LiuHao_T@shu.edu.cn (H.L.); LonggoingTan@i.shu.edu.cn (L.T.);; 2 Electronics Materials and Systems Laboratory, Department of Microtechnology and Nanoscience, Chalmers University of Technology, SE-41296 Gothenburg, Sweden; kjell.jeppson@chalmers.se; 3 Key Laboratory of Advanced Display and System Applications, Ministry of Education, Shanghai University, Yanchang Road, Shanghai 200072, China; bwei@shu.edu.cn

**Keywords:** graphene, chemical vapor deposition (CVD), transfer, heater, resistance, heating/cooling rates

## Abstract

In many applications like sensors, displays, and defoggers, there is a need for transparent and efficient heater elements produced at low cost. For this reason, we evaluated the performance of graphene-based heaters with from one to five layers of graphene on flexible and transparent polyethylene terephthalate (PET) substrates in terms of their electrothermal properties like heating/cooling rates and steady-state temperatures as a function of the input power density. We found that the heating/cooling rates followed an exponential time dependence with a time constant of just below 6 s for monolayer heaters. From the relationship between the steady-state temperatures and the input power density, a convective heat-transfer coefficient of 60 W·m^−2^·°C^−1^ was found, indicating a performance much better than that of many other types of heaters like metal thin-film-based heaters and carbon nanotube-based heaters.

## 1. Introduction

Transparent resistive heaters were proposed for a variety of applications, such as sensors [1], displays [2], defoggers [3], and defrosters [4]. For certain applications, films of indium tin oxide (ITO) are commonly used materials for transparent heaters; however, poor stretchability and a complicated and costly fabrication process limit their usage [5]. Much effort was devoted to developing replacement materials, with some examples being silver nanowires [6,7,8], carbon nanotube films [9,10], and hybrid composites [11].

The electrothermal properties of graphene, an atom-thick planar sheet of *sp*^2^-bonded carbon atoms in a honeycomb pattern [12,13,14], with superconductivity recently observed in magic-angle graphene superlattices [15], indicate that this two-dimensional (2D) material could be the perfect material for many applications including transparent heater applications. Consequently, graphene-based heaters were recently proposed with graphene obtained by chemical vapor deposition (CVD) [3], from reduced graphene oxide [16,17,18], and from graphene aerogels [19]. Among these methods, CVD appears to be the most attractive for industrial production of graphene because of its scalability [20]. Graphene-based heaters fabricated by CVD are often doped with AuCl_3_, Au-CH_3_NO_2_, or HNO_3_ to enhance their electrothermal performance [21]. However, dopants introduced into graphene films might affect the stability of the material by reacting with ambient molecules, thereby causing material properties to degrade over time.

In this article, we present the results of a study of the electrothermal properties of transparent undoped few-layer graphene-based heaters where from one to five layers of graphene grown by CVD were transferred to flexible polyethylene terephthalate (PET) substrates. Our observations of their heating/cooling rates at different power densities are reported in the upcoming sections.

## 2. Fabrication and Evaluation of Graphene-Based Heater Samples

For the set of experiments presented in this work, monolayer graphene was grown on copper foils under low partial pressure by CVD following a standard procedure previously described in detail [22]. In short, this process involves a ~15-min temperature ramp-up to 1000 °C in ambient argon (1000 sccm)/hydrogen (80 sccm), a 5-min annealing at the growth temperature, a 5-min growth period using a methane precursor (5 sccm), and a ~35-min cool-down to room temperature (RT) again in ambient argon/hydrogen.

After growth, a standard layer-by-layer approach was employed to transfer the graphene from the copper foil onto PET substrates involving spin-coated poly(methyl methacrylate) (PMMA). By repeating the transfer process, a set of samples with between one and five layers of graphene was obtained. Samples were then turned into heaters by deposition of Cr/Au electrodes with a thickness of 10/70 nm at the edges of the graphene on PET samples. Finally, T-type thermocouples were attached to the back side of the PET substrates for in situ monitoring of the heater temperature by using a Keysight 34,970 A data acquisition/data logger switch unit. The accuracy of the thermocouple was estimated to be ±0.5 °C. All measurements were performed in a fume cupboard.

**Figure 1:**
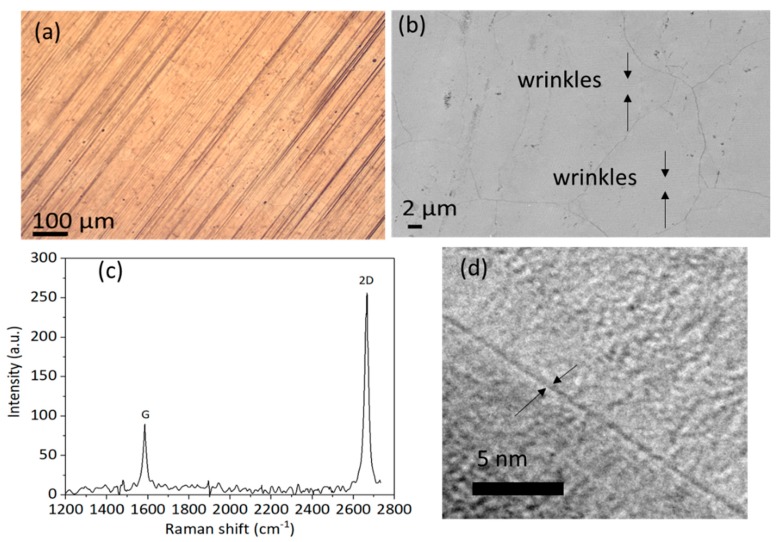
(**a**) Optical image of the morphology of Cu foil after graphene growth. (**b**) SEM image of the morphology of Cu foil after graphene growth. (**c**) Representative Raman spectrum (632 nm) of the graphene grown on Cu foil. (**d**) TEM images of synthesized graphene on Cu foil.

After fabrication, the quality of the graphene was investigated. Optical images showing the morphology of the Cu foil after graphene growth are shown in Figure 1a. As seen using an optical microscope, grain boundaries up to several hundred micrometers long became visible on the Cu foil. These grain boundaries were more clearly identified in scanning electron microscope (SEM) photos, such as the one shown in Figure 1b. The wrinkles were due to the mismatch between the coefficients of thermal expansion of graphene and the underlying metal [23]. It should be noted that those wrinkles crossed the Cu grain boundaries, and that no islands were observed, indicating that the as-grown graphene film was continuous [24]. From the Raman spectrum (Raman measurements were carried out with an XploRA (HORIBA, Ltd. Kyoto, Japan) at a 638-nm excitation wavelength and a 100× objective, with an incident power of ~1 mW) in Figure 1c, typical 2D/G peak intensity ratios of ~2.9 were identified indicating monolayer graphene [25]. As no D band was observed, the Raman spectra suggested as-grown graphene of high quality [22]. The transmission electron microscopy (TEM) image in Figure 1d clearly indicates edges of monolayer graphene, consistent with the Raman spectrum.

**Figure 2:**
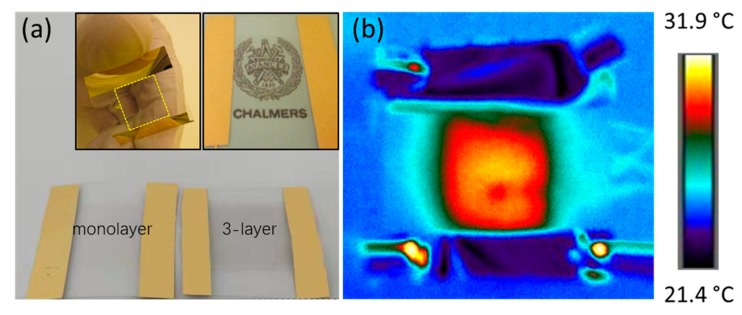
(**a**) Optical images of monolayer and three-layer graphene-based heater (top left inset: a monolayer graphene-based heater for illustrating flexibility; top right inset: a monolayer graphene-based heater placed on a Chalmers logo to illustrate transparency). (**b**) Typical infrared image showing the temperature distribution across the surface of a monolayer graphene heater. The infrared camera used was a high-resolution FLIR A655sc featuring a 640 × 480 pixel microbolometer that can detect temperature differences down to less than 30 mK.

**Figure 3:**
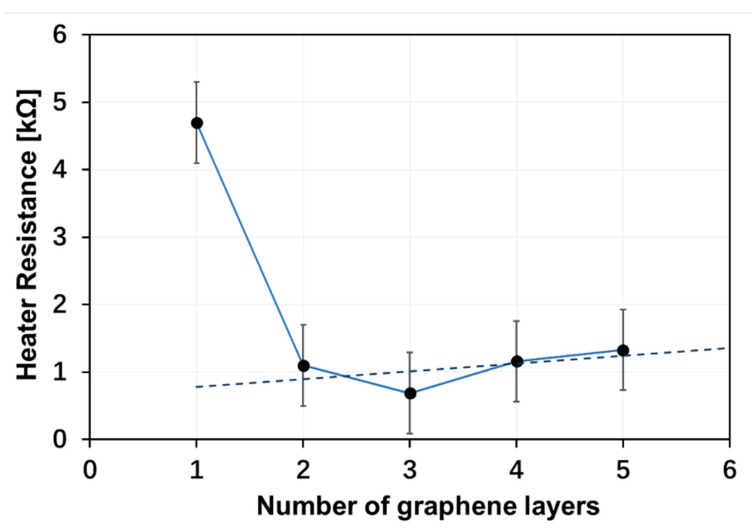
The resistance of the graphene-based heaters versus the number of graphene layers measured using the four-point probe method.

The flexibility and transparency of a monolayer graphene heater sample with an active heating area of ~1.5 × 1.5 cm^2^ is shown in Figure 2a. For comparison, a three-layer graphene heater is shown on the same white background, where it can be seen that the transparency changed in the center area. A transmittance of ~97.7% was reported for monolayer graphene [26]. The corresponding transmittances were ~95.4% for bilayer graphene, ~92.7% for three-layer graphene, ~90% for four-layer graphene, and ~87.3% for five-layer graphene. As shown in Figure 2b, the uniform surface temperature distribution, as obtained by infrared imaging, indicated a uniform graphene film well in line with the SEM image in Figure 1b. The resistance of a set of few-layer graphene-based heaters was evaluated using four-point probing. As shown in Figure 3, the monolayer resistance was close to 5 kΩ, while the resistance of the two- to five-layer graphene heaters was in the 1–1.5-kΩ range. The resistances of the four and five-layer graphene-based heaters appeared to be larger than that of the three-layer graphene heater, which could possibly be explained by the uncertainty of the transfer process causing wrinkles or cracks in the graphene film, or from PMMA residues left from the wet transfer process despite a careful rinse process being used.

## 3. Electrothermal Performance of Graphene-Based Heaters

**Figure 4:**
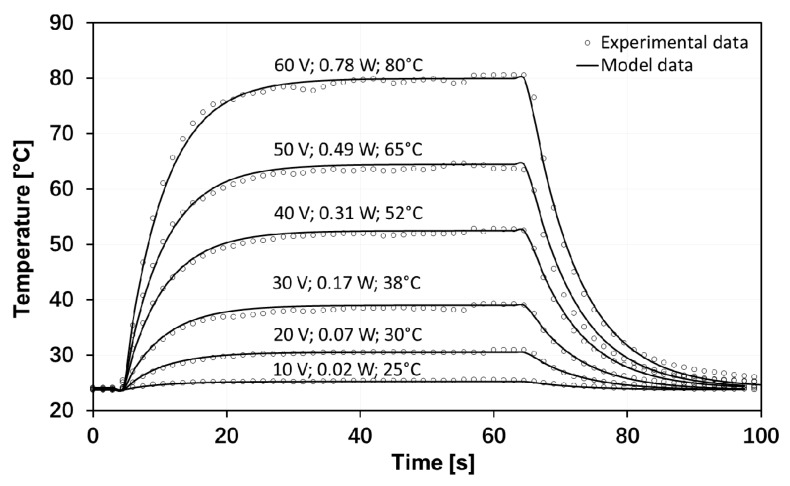
Time dependency of the electrothermal performance of a monolayer graphene-based heater for six different applied input voltages. The numbers occurring next to curves denote applied voltage, input power, and steady-state temperature, respectively. The temperature was logged using T-type thermocouples attached to the back side of the polyethylene terephthalate (PET) substrate.

The heating mechanism of the graphene heaters was Joule heating. The electrothermal performance of monolayer graphene-based heaters determined using T-type thermocouples is shown in Figure 4, showing the heating and cooling behavior for six different values of applied input power. As shown in the figure, the heating and cooling behavior of the graphene-based heaters showed an exponential time dependence with a thermal time constant of 7 s. This time constant indicated the elapsed time required for the temperature difference of the graphene heater to rise to 63% of its final value during heating or, correspondingly to decay to 37% during cooling. It can also be interpreted as the time it would have taken to reach the final value if the heating/cooling continued at its initial rate. The heating and cooling behavior of the graphene heaters with other numbers of graphene layers showed a similar exponential time dependence, but with different time constants. As an example, the time constant for five-layer graphene heaters was 14 s during cooling and 10 s during heating. This difference between the heating and cooling rates may be attributed to the temperature-dependent electrical conductivity of graphene [27]. The model equation during cooling can be written as follows:

(1) $$T=RT+\Delta Te^{-\left(t-t_{0}\right)/\tau },$$

where RT is the room temperature, ∆T the temperature difference between room temperature and steady-state temperature, τ is the time constant, and t_0_ is the time when the cooling starts.

**Figure 5:**
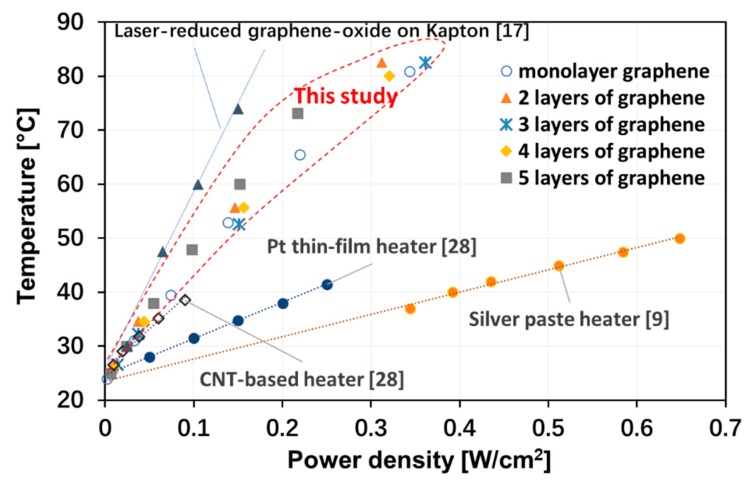
Steady-state temperatures vs. dissipated power density for graphene-based heaters with one, two, three, four, and five layers of graphene. Also shown for comparison are the same temperature versus power density relationships for two metallic heaters and one heater based on carbon nanotube films [9,17,28].

From these experimental plots of the heating/cooling behavior of the graphene heaters, we could also extract the steady-state temperatures versus the input heating power. As shown in Figure 4, a steady-state temperature of 38 °C was obtained for an input power of 170 mW, while a steady-state temperature of 80 °C was obtained for an input power of 780 mW. The results are shown in Figure 5, where the steady-state temperatures were plotted as a function of the power density obtained by dividing the electrical input power by the 2.25-cm^2^ area of the graphene heater. The choice of plotting versus the power density was done to enable a comparison between the performance of our graphene heater and other heaters previously reported in the literature. The plot in Figure 5 shows that graphene-based heaters, for the same input power density, reach much higher temperatures than, for instance, metal-based heaters [8,23]. For low temperatures and limited input power, heaters based on single-walled carbon nanotubes (CNTs) appear comparable, but there are no data available for higher temperatures [28]. Although details of the experimental set-ups may be different, it is obvious that the electrothermal performance of the graphene heaters in this study is much better than the performance of metal-based heaters and somewhat better than that of CNT-based heaters. The electrothermal performance of a laser-reduced graphene oxide heater [17] was even better than this work. It also seems to be a general trend that nanoscale carbon-based heaters electrothermally outperform heaters based on metallic films. However, the most important feature of the graphene-based heaters, making it worthwhile to investigate their performance for potential use in future applications, is their transparency.

**Figure 6:**
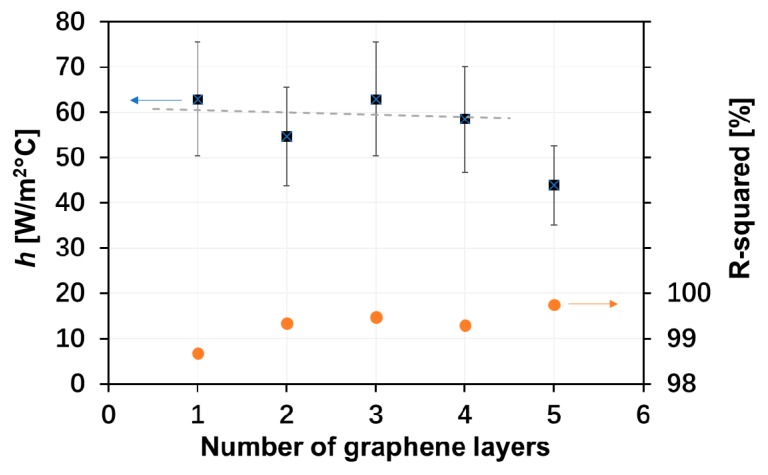
Convective heat-transfer coefficients for graphene-based heaters with from one to five layers of graphene. Also shown are the *R*^2^ regression numbers of the trendline approximations.

From the data in Figure 5, the convective heat-transfer coefficient, *h*, of the graphene heaters could be determined from the trendline slopes of the data for each type of graphene heater, with a calculation method similar to a previous report [4]. Theoretically, the steady-state temperature of a heater is determined by a balance between the electrical input power and the heat loss (mainly due to radiation and convection). For graphene, with its low emissivity, we can neglect the radiation loss and express the convection heat loss by the following equation:

*Q*/*A* = *h* Δ*T*, (2)

where Q/A is the input power density, and Δ*T* = *T* − *T*_0_ is the temperature difference between the steady-state temperature, *T*, and the ambient temperature, *T*_0_. The resulting heat transfer coefficients are shown in Figure 6. There is no obvious trend for the dependence of the heat-transfer coefficient on the number of graphene layers, except possibly for the five-layer graphene heater that showed a somewhat lower value. For this reason, in Figure 5, only an average trendline is shown for the one- to four-layer graphene-based heaters (*h* ≈ 60 W·m^−2^·°C^−1^). Similarly, the Pt thin-film heater had a heat transfer coefficient of ~150 W·m^−2^·°C^−1^, while the corresponding value for the silver paste heater was ~240 W·m^−2^·°C^−1^.

The heat-transfer coefficients obtained for the graphene-based heaters were higher than the coefficient for natural convection of air (~5–25 W·m^−2^·°C^−1^), but in the range for that of forced convection of air (~20–200 W·m^−2^·°C^−1^). The higher value obtained here may be due to our experiments being conducted in a fume cupboard. Deviations between graphene samples were not appreciable, thereby validating the reliability of our measurements. The differences in heat-transfer coefficients between carbon-based heaters and metal thin-film heaters may be attributed to differences in the thermal interface conductance between the solid-gas adsorbates.

**Figure 7:**
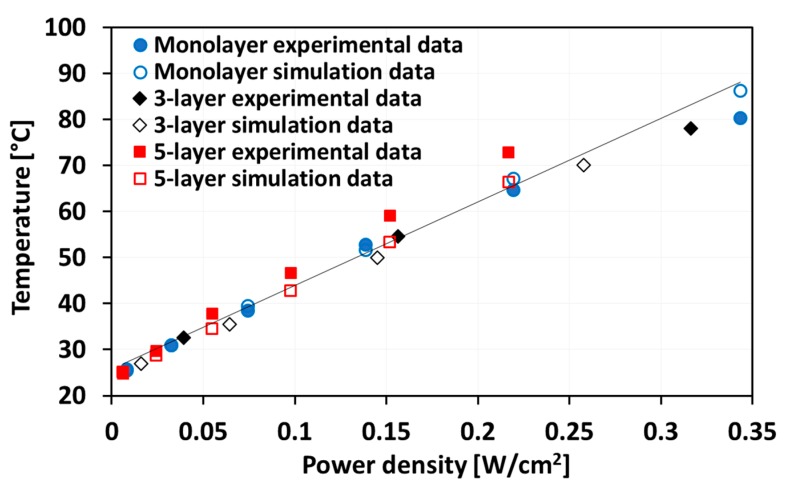
Simulated (open symbols) and experimental (solid symbols) steady-state surface temperatures for monolayer, three-layer, and five-layer graphene-based heaters.

**Figure 8:**
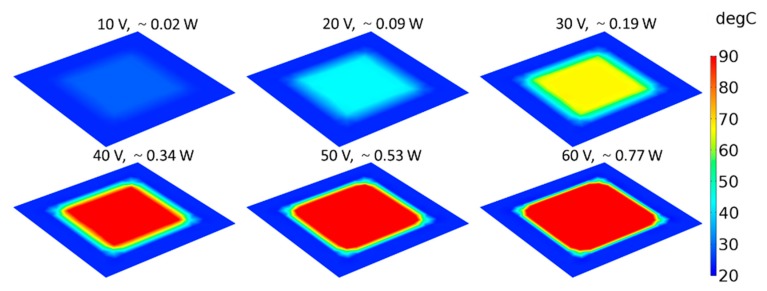
Steady-state surface temperature distributions of monolayer graphene-based heaters for six different applied voltages (the applied powers were also calculated).

Finite element models were developed in COMSOL for studying the steady-state properties of graphene-based heaters and their surface temperature distributions. The electrically generated heat was modeled by using the electric currents and layered shell interface aimed at computing currents and potential distributions in thin conducting layers. Simulations using COMSOL for modeling properties of graphene were reported in previous work [29]. In this work, great care was taken to design the geometry of the model so that it would match the experimental behavior. For different electrical input power and the corresponding potential distributions across the heater surface, simulations resulted in an elevated temperature distribution across the surface of the PET substrate. For comparison between simulations and experiments, where the substrate temperatures were measured by a thermocouple, the average temperature of the backside of PET substrate at different input power densities was also calculated. A comparison between simulations and experiments is shown in Figure 7, where the simulation results show good agreement with the experimental results. What is not visible from the graph in Figure 7 is that, for the same power density, only half the applied voltage was needed for the five-layer graphene-based heater compared to the monolayer heater for the same input power due to the factor of four in their resistance difference. Moreover, the simulated temperature distribution is shown in Figure 8, where it can be seen that the steady-state surface temperature increased with the input voltage. Other graphene heaters had the same trend, whereby the steady-state surface temperature increased with the input voltage, but the values were different.

Finally, an interesting observation made during our experiments was that the resistance of the graphene-based heaters increased after some time of exposure to air. We found that the resistance of the transferred graphene films was significantly increased after a one-year exposure to air, with increases for some samples as much as three to five times, which is much larger than reported in a previous study [30]. Other studies also reported that the resistance of transferred graphene films increased after storage in a humid environment [31,32]. As previously discussed, cracks and residues cannot be completely avoided during the transfer process from the copper foil on which the graphene is grown to the transparent PET substrate. Therefore, there is a risk that water molecules permeate the cracks and traverse the residues, which may weaken the graphene adhesion to the PET substrate and cause an increase in the resistances. It was also found that, when the PET substrates were placed on ice, the resistances of the graphene film increased by 10–20%. However, the resistance returned to the initial values after drying. The increase in resistance indicates that the adhesion between the graphene and the substrate plays a key role in the electrothermal performance of graphene-based heaters in real-world applications.

## 4. Conclusions

Based on the assumption that graphene, with its excellent electrothermal properties, could be a perfect material for transparent heaters in many applications, we designed and fabricated a set of graphene-based heaters with from one to five layers of CVD graphene grown on copper and transferred to a PET substrate. The properties of these graphene-based heaters were evaluated both experimentally and theoretically by simulations in terms of steady-state temperatures and in terms of heating/cooling rates versus the applied power density. In conclusion, we find our results promising in terms of quantifiable parameters such as thermal time constants, maximum heating/cooling rates, and convective heat-transfer coefficients when compared to Ag and Pt metal-based thin-film heaters. However, much work remains to refine the fabrication process and to improve the quality of the graphene films. As an example, we found that the quality of the graphene film and its adhesion to the PET substrate play a key role in determining the performance and reliability of graphene-based heaters when it comes to their electrothermal properties. The results presented here in this study strengthen our belief in graphene-based heaters as being promising candidates for the next generation of transparent heaters for various applications, such as anti-fog windows, mirror defoggers, and outdoor displays.

## References

[1] Mo Y.W., Okawa Y., Tajima M., Nakai T., Yoshiike N., Natukawa K. (2001). Micro-machined gas sensor array based on metal film micro-heater. Sens. Actuat. B Chem..

[2] Liu P., Liu L.A., Jiang K.L., Fan S.S. (2011). Carbon-Nanotube-film microheater on a polyethylene terephthalate substrate and its application in thermochromic displays. Small.

[3] Bae J.J., Lim S.C., Han G.H., Jo Y.W., Doung D.L., Kim E.S., Chae S.J., Huy T.Q., Luan N.V., Lee Y.H. (2012). Heat dissipation of transparent graphene defoggers. Adv. Funct. Mater..

[4] Kiruthika S., Gupta R., Kulkarni G.U. (2014). Large area defrosting windows based on electrothermal heating of highly conducting and transmitting Ag wire mesh. RSC Adv..

[5] Chuang M.J. (2010). ITO films prepared by long-throw magnetron sputtering without oxygen partial pressure. J. Mater. Sci. Technol..

[6] De S., Higgins T.M., Lyons P.E., Doherty E.M., Nirmalraj P.N., Blau W.J., Boland J.J., Coleman J.N. (2009). Silver nanowire networks as flexible, transparent, conducting films: Extremely high DC to optical conductivity ratios. ACS Nano.

[7] Celle C., Mayousse C., Moreau E., Basti H., Carella A., Simonato J.P. (2012). Highly flexible transparent film heaters based on random networks of silver nanowires. Nano Res..

[8] Ji S.L., He W.W., Wang K., Ran Y.X., Ye C.H. (2014). Thermal response of transparent silver nanowire/PEDOT:PSS film heaters. Small.

[9] Yoon Y.H., Song J.W., Kim D., Kim J., Park J.K., Oh S.K., Han C.S. (2007). Transparent film heater using single-walled carbon nanotubes. Adv. Mater..

[10] Jang H.S., Jeon S.K., Nahm S.H. (2011). The manufacture of a transparent film heater by spinning multi-walled carbon nanotubes. Carbon.

[11] Kim D., Zhu L.J., Jeong D.J., Chun K., Bang Y.Y., Kim S.R., Kim J.H., Oh S.K. (2013). Transparent flexible heater based on hybrid of carbon nanotubes and silver nanowires. Carbon.

[12] Bolotin K.I., Sikes K.J., Jiang Z., Klima M., Fudenberg G., Hone J., Kim P., Stormer H.L. (2008). Ultrahigh electron mobility in suspended graphene. Solid State Commun..

[13] Prasher R. (2010). Graphene spreads the heat. Science.

[14] Pang S.P., Hernandez Y., Feng X.L., Mullen K. (2011). Graphene as transparent electrode material for organic electronics. Adv. Mater..

[15] Cao Y., Fatemi V., Fang S., Watanabe K., Taniguchi T., Kaxiras E., Jarillo-Herrero P. (2018). Unconventional superconductivity in magic-angle graphene superlattices. Nature.

[16] Sui D., Huang Y., Huang L., Liang J.J., Ma Y.F., Chen Y.S. (2011). Flexible and transparent electrothermal film heaters based on graphene materials. Small.

[17] Romero F.J., Rivadeneyra A., Ortiz-Gomez I., Salinas A., Godoy A., Morales D.P., Rodriguez N. (2019). Inexpensive graphene oxide heaters lithographed by laser. Nanomaterials.

[18] Bobinger M.R., Romero F.J., Salinas-Castillo A., Becherer M., Lugli P., Morales D.P., Rodriguez N., Rivadeneyra A. (2019). Flexible and robust laser-induced graphene heaters photothermally scribed on bare polyimide substrates. Carbon.

[19] Menzel R., Barg S., Miranda M., Anthony D.B., Bawaked S.M., Mokhtar M., Al-Thabaiti S.A., Basahel S.N., Saiz E., Shaffer M.S.P. (2015). Joule heating characteristics of emulsion-templated graphene aerogels. Adv. Funct. Mater..

[20] Bae S., Kim H., Lee Y., Xu X., Park J.S., Zheng Y., Balakrishnan J., Lei T., Kim H.R., Song Y.I. (2010). Roll-to-roll production of 30-inch graphene films for transparent electrodes. Nat. Nanotechnol..

[21] Kang J., Kim H., Kim K.S., Lee S.K., Bae S., Ahn J.H., Kim Y.J., Choi J.B., Hong B.H. (2011). High-performance graphene-based transparent flexible heaters. Nano Lett..

[22] Gao Z., Zhang Y., Fu Y., Yuen M.M.F., Liu J. (2013). Thermal chemical vapor deposition grown graphene heat spreader for thermal management of hot spots. Carbon.

[23] Zhang Y., Fu Y., Edwards M., Jeppson K., Ye L., Liu J. (2017). Chemical vapor deposition grown graphene on Cu-Pt alloys. Mater. Lett..

[24] Li X., Cai W., An J., Kim S., Nah J., Yang D., Piner R., Velamakanni A., Jung I., Tutuc E. (2009). Large-area synthesis of high-quality and uniform graphene films on copper foils. Science.

[25] Ferrari A.C., Meyer J.C., Scardaci V., Casiraghi C., Lazzeri M., Mauri F., Piscanec S., Jiang D., Novoselov K.S., Roth S. (2006). Raman spectrum of graphene and graphene layers. Phys. Rev. Lett..

[26] Nair R.R., Blake P., Grigorenko A.N., Novoselov K.S., Booth T.J., Stauber T., Peres N.M.R., Geim A.K. (2008). Fine structure constant defines visual transparency of graphene. Science.

[27] Fang X.Y., Yu X.X., Zheng H.M., Jin H.B., Wang L., Cao M.S. (2015). Temperature- and thickness-dependent electrical conductivity of few-layer graphene and graphene nanosheets. Phys. Lett. A.

[28] Kang T.J., Kim T., Seo S.M., Park Y.J., Kim Y.H. (2011). Thickness-dependent thermal resistance of a transparent glass heater with a single-walled carbon nanotube coating. Carbon.

[29] Subrina S., Kotchetkov D., Balandin A.A. (2009). Heat removal in silicon-on-insulator integrated circuits with graphene lateral heat spreaders. IEEE Electr. Device Lett..

[30] Tan L.F., Zeng M.Q., Wu Q., Chen L.F., Wang J., Zhang T., Eckert J., Rummeli M.H., Fu L. (2015). Direct growth of ultrafast transparent single-layer graphene defoggers. Small.

[31] Hwang J., Kim M., Campbell D., Alsalman H.A., Kwak J.Y., Shivaraman S., Woll A.R., Singh A.K., Hennig R.G., Gorantla S. (2013). van der Waals epitaxial growth of graphene on sapphire by chemical vapor deposition without a metal catalyst. ACS Nano.

[32] Jung W., Park J., Yoon T., Kim T.S., Kim S., Han C.S. (2014). Prevention of water permeation by strong adhesion between graphene and SiO_2_ substrate. Small.

